# The Impact of Mitochondrial Dysfunction in Amyotrophic Lateral Sclerosis

**DOI:** 10.3390/cells11132049

**Published:** 2022-06-28

**Authors:** Jiantao Zhao, Xuemei Wang, Zijun Huo, Yanchun Chen, Jinmeng Liu, Zhenhan Zhao, Fandi Meng, Qi Su, Weiwei Bao, Lingyun Zhang, Shuang Wen, Xin Wang, Huancai Liu, Shuanhu Zhou

**Affiliations:** 1 Department of Histology and Embryology, School of Basic Medical Sciences, Weifang Medical University, Weifang 261053, China; zjt15233694339@163.com (J.Z.); 18863662168@163.com (X.W.); zijunhuo1998@163.com (Z.H.); cyc7907@wfmc.edu.cn (Y.C.); 15069327395@163.com (Z.Z.); 18800460605@163.com (F.M.); suqi5976@163.com (Q.S.); jssybaoweiwei96@163.com (W.B.); 2 Neurologic Disorders and Regenerative Repair Laboratory, Weifang Medical University, Weifang 261053, China; 18353687185@163.com (J.L.); zly199311@126.com (L.Z.); 3 Department of Joint Surgery, Affiliated Hospital of Weifang Medical University, School of Clinical Medicine, Weifang Medical University, Weifang 261061, China; ws18853696596@163.com; 4 Department of Neurosurgery, Brigham and Women’s Hospital, Harvard Medical School, Boston, MA 02115, USA; xwang@rics.bwh.harvard.edu; 5 Department of Orthopedic Surgery, Brigham and Women’s Hospital, Harvard Medical School, Boston, MA 02115, USA

**Keywords:** amyotrophic lateral sclerosis, mitochondrial dysfunction, neurodegenerative diseases

## Abstract

Amyotrophic lateral sclerosis (ALS) is a rapidly progressive and highly fatal neurodegenerative disease. Although the pathogenesis of ALS remains unclear, increasing evidence suggests that a key contributing factor is mitochondrial dysfunction. Mitochondria are organelles in eukaryotic cells responsible for bioenergy production, cellular metabolism, signal transduction, calcium homeostasis, and immune responses and the stability of their function plays a crucial role in neurons. A single disorder or defect in mitochondrial function can lead to pathological changes in cells, such as an impaired calcium buffer period, excessive generation of free radicals, increased mitochondrial membrane permeability, and oxidative stress (OS). Recent research has also shown that these mitochondrial dysfunctions are also associated with pathological changes in ALS and are believed to be commonly involved in the pathogenesis of the disease. This article reviews the latest research on mitochondrial dysfunction and its impact on the progression of ALS, with specific attention to the potential of novel therapeutic strategies targeting mitochondrial dysfunction.

## 1. Introduction

Amyotrophic lateral sclerosis is a complex and common multipathogenic neurodegenerative disease, characterized by the progressive loss of upper and lower motor neurons (MNs). Progressive degeneration of the limbs leads to muscle atrophy and paralysis, and finally, death (mainly due to respiratory failure) 3 to 5 years after onset. The incidence of ALS is 0.6 to 3.8 per 100,000 people worldwide, with notable differences between ethnic groups [1]. From a genetic point of view, there are two main types of ALS: familial ALS (fALS) and sporadic ALS (sALS). Roughly 90% of cases are sporadic without an associated genetic cause, and 10% are related to family history and dominant inheritance [2]. The age of onset of ALS is between 55 and 65 years, but the onset of fALS is earlier [3]. In familial cases, 25% of cases survive on average 2 to 3 years, and though some can survive up to 5 years, only 5–10% survive to 10 years [4]. There is currently no cure for ALS, and no drugs have been shown to be effective against the disease. Although two drugs, riluzole and edaravone, have been approved for marketing [5,6,7], they only delay functional loss and prolong survival by several months [8]. Based on the current understanding of the pathogenesis of the disease and the effects of clinical treatments, by the point of diagnosis, the pathogenic cascade of ALS may have matured, and the degeneration of neurons has already occurred [9]. Since the discovery of superoxide dismutase 1 (SOD1), the first gene associated with ALS in 1993, more than 20 other genes have been found to be causally or highly correlated with its pathogenesis, including transactive response DNA binding protein 43 kDa (TDP-43) [10], fused in sarcoma/transfer in liposarcoma (FUS), matrin 3 (MATR3), coiled-coil-helix-coiled-coil-helix domain containing 10 (CHCHD10), tank-binding kinase 1 (TBK1), tubulin, alpha 4A (TUBA4A), chromosome 21 open reading frame 2 (C21orf2), chromosome 9 open reading frame 72 (C9orf72), and cyclin F (CCNF), among others. These genes encode one or more molecular pathways, and their proteins were identified by genome-wide association studies, genome-wide studies, or exome sequencing methods [11]. Even so, our understanding of the pathogenesis of ALS is still in its infancy, and current research focuses on protein aggregation and misfolding, OS [12], neuroinflammation, epigenetics, and mitochondrial dysfunction. It is precisely because of the critical role of mitochondria in maintaining cellular homeostasis that current research is focused largely on mitochondria, and more and more findings support the idea that mitochondrial dysfunction plays an active role in ALS pathogenesis [13].

Better research and understanding of disease pathogenesis (which may be multifactorial) and the identification of novel biomarkers and phenotypic modifications will lead to a better subclinical classification of disease and the development of targeted drugs. Similarly, it will facilitate the development of better prognostic criteria, and interventions in the disease process will be more effective. In general, factors such as genetic mutations [14] and weakened autoimmunity are among the many factors that contribute to the death of MNs. Current evidence suggests that the innate immune system plays a role in ALS. The release of pro-inflammatory cytokines has been shown to lead to motor neuron damage [15]. Some pathological changes may have already begun before there is any obvious movement disorder, and many factors leading to the death of MNs are directly or indirectly aggregated in mitochondria. In this review, we investigate the impact of mitochondrial dysfunction in ALS and how current research could yield potentially effective interventions and treatments during disease progression.

## 2. The Main Function of Mitochondria

Mitochondria, as organelles of almost all eukaryotes, are composed of four-part structures and are involved in the regulation of many cellular functions, such as ATP production, Ca^2+^ signaling, maintenance of cellular homeostasis, and apoptosis. The dysregulation of these mitochondrial functions can lead to the development of disease [16]. Once the cells are stimulated by the external environment, energy needs can be matched by increasing mitochondrial mass. Energy supply can be increased by processes that increase the mass of individual mitochondria, as stimulated by exercise training, electrical stimulation, hormones, etc. [17].

In addition, mitochondria also maintain, repair, and reorganize themselves through fusion and fission. At the same time, in order to meet the energy needs of the body, mitochondria will also increase energy output through fusion and other methods, increasing the level of oxidative phosphorylation (OXPHOS).

Mitochondria are the main site of OXPHOS and metabolism. Their main function is to generate ATP for cells, which is essential for the function of energy-demanding organs such as the brain and heart. Therefore, these organs are most vulnerable to changes in mitochondrial energy supply [18]. In addition, mitochondria produce reactive oxygen species (ROS) even under basal energy demands, and approximately 90% are produced during the process of OXPHOS [19]. Excessive ROS production causes abnormal OS in vivo, which plays a significant role in the onset and development of ALS. In this process, reducing molecules such as nicotinamide adenine dinucleotide (NADH) and reduced flavin adenine dinucleotide (FADH2) (reduced analogs of the cytoplasmic substrate) can enter the electron transport chain (ETC) from the malate-aspartate transport system or the phosphoglycerol transport system. After several steps in the ETC, oxygen is finally reduced and energy is released; some is used to generate ATP, and the rest dissipates as heat. The mitochondrial complex consists of five complexes on the IMM, which undergo a series of redox processes and finally form ATP. Similarly, the mitochondrial complex is also one of the main energy supply methods of the body. Enzyme complexes on the inner mitochondrial membrane (IMM), including NADH-ubiquinone reductase, ubiquinone-cytochrome C reductase, and cytochrome C oxidase, use the energy released to pump protons into the mitochondrial intermembrane space against the concentration gradient. Although the process is efficient, a small number of electrons prematurely reduce oxygen to form ROS such as superoxide, which can degrade mitochondrial performance. When protons are pumped into the mitochondrial intermembrane space, an electrochemical gradient is established on both sides of the IMM, and protons tend to diffuse along the concentration gradient. ATP synthase (respiratory chain complex V) has a proton channel and an ATP synthesis site. Driven by the concentration gradient, the protons on both sides of the inner membrane twitch the rotation of the ATP synthesis site through the proton channel to synthesize ATP from ADP and Pi. This process is performed by the other four complexes and OXPHOS. The inhibition or deficiency of mitochondrial respiratory chain complex activity can lead to severe mitochondrial dysfunction, and long-term inhibition or deficiency can lead to neurological diseases such as ALS, Parkinson’s disease, Down’s syndrome, and Leigh’s syndrome, etc. Therefore, mitochondrial respiratory chain complex activity is often used as an indicator of neurodeficient diseases caused by mitochondrial dysfunction.

In addition to calcium storage, mitochondria also coordinate the functions of structures such as the endoplasmic reticulum and the extracellular matrix to control the homeostasis of intracellular calcium concentrations. Not only are there many Ca^2+^ targets on the mitochondrial surface, but the mitochondrial transport of Ca^2+^ also regulates many aspects of mitochondrial function [20]. Ca^2+^ uptake is an electrogenic process mediated by the selective Ca^2+^ ion channel, the mitochondrial Ca^2+^ monotransporter (MCU) [21,22]. However, Ca^2+^ uptake results in only a low affinity for the entire complex because of a threshold in the presence of low cytoplasmic Ca^2+^ concentrations [23]. Even so, the cytoplasmic Ca^2+^ signal is rapidly switched into the mitochondrial matrix. The high efficiency of this process can be attributed to the tight binding between mitochondria and Ca^2+^ hotspots, and the ability of mitochondria to rapidly absorb calcium ions makes them a buffer for calcium ions in cells [24].

Mitochondrial dynamics are regulated by fusion and fission and are involved in cellular stress responses and energy production. It is also a key component in maintaining mitochondrial homeostasis and regulating mitochondrial function. Mitochondrial fusion proteins include Mfn1 and Opa1, and fission proteins include Fis1 and Drp1. The extent of mitochondrial networking is the result of a dynamic balance between fusion and fission promoted by mitochondrial movement within the cell. In general, more networked mitochondria appear to be more efficient at producing ATP, especially through aerobic metabolism [25].

## 3. Mitochondrial Dysfunction and ALS

**Figure 1:**
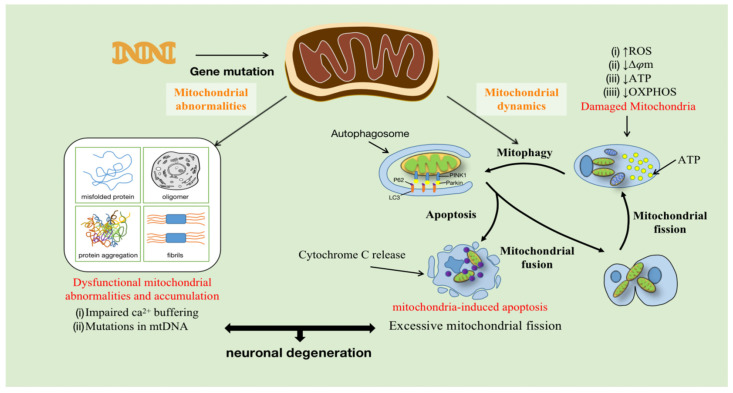
Mitochondrial genes such as FUS, SOD1, and CHCHD10 are mutated in ALS patients, leading to mitochondrial damage. During mitochondrial fission, OXPHOS decreases due to impaired mitochondria, which results in decreased ATP production, decreased transmembrane potential (Δψm), and increased ROS production, while damaged mitochondrial components are eliminated by autophagy. Furthermore, higher mitochondrial membrane permeability leads to increased release of cytochrome C, promoting the production of more pro-apoptotic proteins. Mutations in mitochondrial DNA (mtDNA) and excessive mitochondrial shedding lead to synaptic dysfunction, axonal degeneration, and ultimately neuronal degeneration and death.

Mitochondria are composed of the OMM (outer mitochondrial membrane), the inter membrane space (IMS), the IMM, and the mitochondrial matrix, which perform multiple functions in cells, including energy production, material metabolism, the synthesis of iron-sulfur clusters, and programmed cell death. Mitochondrial homeostasis is dynamically maintained through processes such as mitochondrial fusion/fission, mitochondrial phagocytosis, and apoptosis. Research on mitochondrial dysfunction in various diseases has revealed that mitochondria are essential for cell development and survival. The activities of the human body, including normal neuronal function, require a large amount of ATP, and mitochondria are the main energy production sites [26]. Furthermore, neuronal mitochondria are key regulators of intracellular Ca^2+^ homeostasis. Mitochondria, together with the endoplasmic reticulum, control dendritic Ca^2+^ levels and buffer Ca^2+^ in presynaptic terminals and axonal bundles to regulate neurotransmission [27,28,29,30,31]. Ca^2+^ transients also control the level of mitochondrial matrix Ca^2+^ to stimulate mitochondrial ATP production [32]. Typically, impaired mitochondrial function is largely caused by damage to the ETC, which affects OXPHOS and ATP production. Interestingly, respiratory chain deficiency has indeed been found in patients with ALS [33]. Damage to the ETC may lead to changes in oxygen consumption, a decreased transmembrane potential (Δψm), and an increased production of ROS, which directly lead to oxidative damage, including reduced ATP synthesis [34]. In addition, inefficient OXPHOS can also generate ROS, causing mitochondrial dysfunction [35]. Therefore, mitochondria are the main source of both ATP and ROS. Once ROS is overproduced, it may lead to OS, impair mitochondrial function (production of misfolded proteins, oligomers, fibrils, and protein aggregates, etc.), and dysregulate mitochondria, leading first to mitophagy and apoptosis, and eventually to cell death [36] (Figure 1).

Numerous potential pathogenic or disease-modifying genes have now been identified, and SOD1 plays an important role in maintaining mitochondrial homeostasis and preventing apoptosis [37]. The SOD1-G93A mutant gene binds with Bcl-2 to form a complex and localizes in mitochondria. Bcl-2 inhibits apoptosis by regulating cytochrome C release and mitochondrial-initiated caspase activation [38,39]. In addition, glutaredoxins (Grxs) can also reduce disulfides to protein thiols that prevent the aggregation of mutant SOD1, preserving mitochondrial function and protecting neuronal cells from apoptosis. However, the overexpression of Grxs1 in IMS may accelerate mitochondrial fragmentation [40]. C9orf72 is a mitochondrial inner membrane-associated protein in which the GGGGCC sequence repeat expansion is a common genetic cause of ALS [41], characterized by the accumulation of dipeptide repeat (DPR)-containing proteins in mitochondria and the formation of aggregates in neurons [42]. Translocase of inner mitochondrial membrane domain containing 1 (TIMMDC1) is an essential component in mitochondrial complex I assembly. C9orf72 regulates oxidative phosphorylation by stabilizing TIMMDC1, but C9orf72 haploinsufficiency and a loss of function lead to the reduced activity of mitochondrial complex I in C9orf72-ALS patient-derived neurons, directly or indirectly causing or exacerbating the disease [43]. The activity of mitochondrial complex I decreases, which directly or indirectly causes or aggravates the disease [44]. Elisa et al. initially found that mitochondrial function in TDP-43 and C9orf72 fibroblasts were affected under oxidative conditions and impaired mitochondrial activity in ALS neurons [45]. Second, Tania et al. demonstrated that the expression of TDP-43 resulted in a dose-dependent decrease in TDP-43 RNA and protein in SOD1-G93A mice, along with increased levels of Fis1 and Drp1, which play important roles in the mitochondrial fission machinery. In contrast, the expression of Mfn1, which plays an important role in mitochondrial fusion, was significantly reduced [46]. Toru et al. found that the expression of the fission proteins Drp1 and Fis1 was high in Tg mice during the first 10 weeks of symptoms, while the expression of fusion proteins Mfn1 and Opa1 was gradually decreased [47]. In summary, their findings suggest that fission proteins such as Fis1 and Drp1 and fusion proteins such as Mfn1 and Opa1 play significant roles in influencing mitochondrial dynamics. When mitochondrial fusion or fission processes are overactive, it leads to excessive mitochondrial fragmentation, which activates apoptosis in cells. It also affects various aspects of mitochondrial function, including ETC, Ca^2+^ homeostasis, ATP, and ROS production, which directly affect the progression of ALS [48].

Mitophagy can timely remove aging and damaged mitochondria, which plays an important role in cell growth. The process of mitophagy is to promote the rupture of the tubular network and the separation of damaged mitochondria after receiving intracellular and extracellular signals, and subsequently recruit receptors on the mitochondrial surface, while generating an isolation membrane around mitochondria, targeting mitochondria to be wrapped by autophagosomes. Then, autophagosomes are transported and fused with the lysis chamber, lysosomes flow into autophagosomes to degrade mitochondria, and finally the degraded contents are recycled. Optineurin (OPTN) [49] has been identified as the primary receptor for PINK1/Parkin-mediated filamentous phagocytosis, which binds ubiquitin chains of damaged mitochondria to induce phagocytosis [50]. While autophagosome phagocytosis of impaired mitochondria is dependent on OPTN and its kinase TBK1, the loss of OPTN or TBK1 function results in impaired mitochondrial phagocytosis and the accumulation of damaged mitochondria [51]. The autophagy-encoding receptor SQSTM1 can interact with LC3 through its LC3-interacting region (LIR) to help degrade ubiquitinated molecules. The SQSTM1/p62 ALS-linked L341V mutation is defective in LC3B recognition, which makes SQSTM1 less susceptible to incorporation into autophagic vesicles [52]. The loss of SQSTM1 deregulates the autophagy-lysosomal degradation system, while mutation of the ALS-linked L341V LIR also reduces the affinity of SQSTM1 for LC3 and delays SQSTM1 degradation in cells [53].

The dysfunction of mitochondria may stem from morphological defects. Studies have shown that C9orf72 regulates energy homeostasis by stabilizing mitochondrial complex I. C9orf72 haploinsufficiency destabilizes mitochondrial complex I and drives motor neuron degeneration. At the same time, studies have also found that when the morphology of mitochondria remains intact, C9orf72 can be protected from damage [44]. In cells extracted from ALS patients, mitochondrial membrane potential and respiratory chain complex activity were both decreased following changes in mitochondrial morphology. In the SOD1-G93A transgenic mouse model and other cell models, when mitochondrial morphology is defective, decreased ATP levels, Ca^2+^ disturbance, and increased ROS generation can be observed [54]. These studies on mitochondrial morphological defects directly or indirectly affect mitochondrial function and become direct evidence of mitochondrial dysfunction in ALS.

However, there are many other causes of mitochondrial dysfunction. In addition to factors such as the excessive production of ROS and the disruption of calcium buffering, factors such as the disruption of axonal transport, mitochondrial structure, dynamics, mitosis, and apoptotic signaling are also disrupted. These factors are the main factors leading to the onset of the disease, which in turn affects the growth and development of neurons [55].

## 4. Mitochondrial Dysfunction and Oxidative Stress in ALS

**Figure 2:**
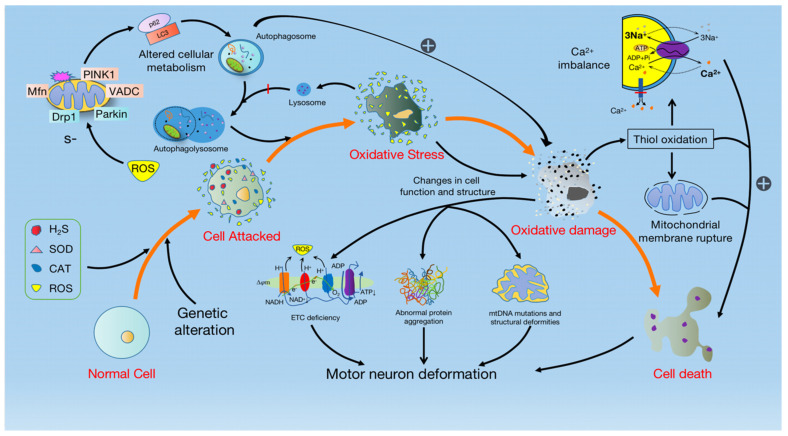
OS triggers mitochondrial dysfunction and leads to MN degeneration. The invasion of abnormal substances such as H2S, SOD, Catalase (CAT), and ROS causes cells to generate OS, resulting in the enhancement of PINK1-Parkin-mediated mitophagy and the accumulation of mitochondrial damage. Simultaneously, mitochondrial damage in turn causes thiol oxidation, triggering the cascading effects of Ca^2+^ imbalance and mitochondrial membrane rupture, which activates cell death. In addition, the impairment of ETC due to mitochondrial damage reduces the production of NAD^+^ and ATP, and increases the production of ROS, resulting in abnormal protein aggregation, mtDNA mutation, and structural deformity, and eventually leads to motor neuron degeneration.

There is increasing evidence that OS is inextricably linked to the pathogenesis of mitochondrial dysfunction and motor neuron degeneration. When normal cells are attacked by abnormal substances such as hydrogen sulfide (H_2_S), SOD1, and catalase (CAT), the activity of the enzyme is altered, causing normal cells to trigger OS. Whereas OS is the result of increased ROS generation, often accompanied by decreased antioxidant defense [56]. Although ROS produced in normal cells is not thought to cause ALS, excessive production may contribute to the development of the disease. Damage caused by oxidative stress can also trigger chain reactions such as abnormal protein aggregation, mtDNA mutations, and ETC mutations. The mutation of the ETC not only leads to increased ROS production, but also exacerbates the degree of mutation of the ETC, and eventually leads to motor neuron degeneration [55] (Figure 2).

### 4.1. Mitochondria as the Main Source of Reactive Oxygen Species

ROS are one-electron reduction products of a class of oxygen that are mainly produced in the mitochondria, peroxisomes, and endoplasmic reticulum of cells through the ETC, cytochrome p-450 activity, prostaglandin synthesis, and phagocytosis [57,58]. Among them, mitochondrial activity and the metabolism of cytochrome p-450 is the main source of ROS in mammalian cells [59]. Mitochondria are one of the most important intracellular sources of ROS. Mitochondria play an important role in the production of oxidative ATP, and molecular oxygen is reduced to water in the process of the ETC [60]. However, mitochondrial dysfunction can lead to damage to the ETC, producing an over reduced state [61,62]. At the same time, damage to the respiratory chain leads to the redundancy and leakage of electrons, which react with oxygen, further increasing the amount of ROS generated [63,64]. Under both normal physiological and pathological conditions, ROS production in mitochondria may occur in the outer membrane, the inner membrane, or the mitochondrial matrix. The activation of immune cells, inflammatory responses, and increased mental stress are all causes of endogenous ROS generation. The generation of exogenous ROS may be due to ingestion or exposure to environmental pollutants, heavy metals, drugs, chemical solvents, second-hand smoke, alcohol, and radiation, among other causes [65]. These exogenous substances are degraded or metabolized after entering the human body, activating the ROS production mechanism, resulting in an increase in ROS production.

### 4.2. Oxidative Stress in ALS Induced by ROS

ROS are generated during normal cellular metabolism and are extremely important for maintaining cellular homeostasis. OS is a state of redox imbalance in which the body’s antioxidant defense capacity is reduced due to increased ROS production [66,67]. However, excess ROS can be detrimental, producing unfavorable oxidative modifications to cellular components, including to the mitochondrial structure, the primary target of ROS-induced damage [68]. At the same time, excessive ROS can also cause different degrees of oxidative damage to intracellular proteins, lipids, DNA, and other components [69]. Pathological mechanisms induced directly or indirectly by ROS can cause neuronal damage and degeneration, especially in the brain [70,71]. The brain is particularly susceptible to OS [72] because of its high oxygen consumption and low antioxidant defenses. In addition, the brain is composed mainly of polyunsaturated fatty acids (PUFAs), which are highly sensitive to lipid peroxide composition [73] and are easily oxidized [74]. Mitochondrial dysfunction impairs the ATP energy supply to neurons and calcium homeostasis, leading to high levels of ROS, accelerated mtDNA mutation rates and the lipid peroxidation of neuronal membranes [56]. In addition, MNs are very sensitive to OS [75], and the central nervous system has a poor antioxidant capacity and a low activity of protective enzymes such as CAT and SOD1, resulting in a low cell regeneration capacity. Thus, ALS progresses irreversibly [76]. Furthermore, the accumulation of mtDNA mutations leads to increased oxidative damage, a decreased energy production, and increased ROS. Thus, mitochondrial dysfunction creates a vicious cycle of neuronal damage, genetic mutation, and metabolic stress, which may lead to apoptosis, which can lead to disease onset or accelerate disease progression [77].

In many neurodegenerative diseases, ROS can lead to mtDNA mutations, a calcium homeostasis imbalance, and an increased membrane permeability [78,79]. Because OS is considered a key pathogenic factor in ALS, hundreds of antioxidant stressors have now been tested for their therapeutic potential. Riluzole, an anti-glutamatergic drug approved by the U.S. Food and Drug Administration in 1995, can delay the progression of the disease and the lifespan of patients to a certain extent, but its efficacy is limited [80]. Studies have reported that riluzole can inhibit glutamate release and glutamatergic transmission in the brain and can attenuate oxidative damage in neuronal cells caused by ALS in SOD1-G93A mice [81,82]. In addition, riluzole has also been reported to increase survival in ALS patients who have undergone a tracheostomy [83]. Increased survival and improved motor function have been reported in patients receiving riluzole [84,85]. Recently, the antioxidant drug edaravone, developed by Mitsubishi Tanabe Pharma, was found to be effective in preventing motor function deterioration in early ALS. The newly approved drug edaravone is a force multiplier for ALS treatment [86]. Mitochondrial abnormalities have been found in spinal cord and muscle anatomical samples from ALS patients, along with defects in mitochondrial respiratory chain complexes and elevated oxidative stress. Meanwhile, a vicious cycle of abnormal ROS signaling and excessive ROS production was also found to significantly promote muscle atrophy in mouse models during ALS progression [87].

### 4.3. Oxidative Stress-Mediated Intracellular Nrf2/Keap1 Signaling Pathway in ALS

ROS can regulate the nuclear factor erythroid 2-related factor 2 (Nrf2)/Keap1 antioxidant response element (ARE) intracellular signaling pathway at the molecular level to promote disease progression [88]. This pathway plays an important role in regulating ROS-induced cellular OS and has a protective effect against neurodegenerative diseases, including ALS [89,90]. It has also been reported that the expression of Nrf2 is significantly reduced in the MNs of SOD1-G93A mice [91]. Another study also reported reduced Nrf2 levels in the motor cortex, the spinal cord, and lower extremity muscle MNs [92]. On the other hand, the deletion of Nrf2 in SOD1-G93A mice accelerated motor neuron death and astrocyte activation, resulting in the early onset of the disease [93]. Such findings show that SOD1 gene mutation leads to the reduction of antioxidant response protein, considered one of the main cytokines that cause ALS pathogenesis.

### 4.4. The Role of Mitochondrial Protein Homeostasis in ALS

The maintenance of mitochondrial proteostasis prevents mitochondrial damage. This protective mechanism ensures the proper hydrolysis and degradation of misfolded, oxidatively damaged, and damaged proteins by proteases within the mitochondria. In addition, proper mitochondrial function can also be maintained by eliminating damaged organelles that cannot be repaired. Mitochondria have their own unfolded protein response (UPR), which is activated when too much misfolded protein accumulates. In ALS, the excessive accumulation of misfolded and oxidatively damaged proteins may slow or even prevent the progression of the UPR [94]. A study of postmortem spinal cords of sporadic and familial ALS patients have found that UPR triggers a reduction in general translation and an increase in the expression of genes encoding chaperones, foldases, and ERAD proteins, but does not restore mitochondrial homeostasis. Furthermore, the cytoplasmic accumulation of TDP-43 may be driven by the activation of motor neuron ER stress. ALS mutations in TDP-43 lead to UPR upregulation in neural 2A cell models. The accumulation of TDP-43 can promote the increase of endoplasmic reticulum stress levels and the subsequent activation of apoptosis [95]. Mitochondria contain numerous proteolytic enzymes to build the mitochondrial proteome, maintain and control its function, or degrade mitochondrial proteins and peptides. OMA1 is a metalloprotease located on the inner membrane, which is dormant under normal conditions and rapidly activates under stressful conditions, such as the loss of membrane potential, heat, or oxidative stress. Studies have shown that under physiological conditions, CHCHD2 and CHCHD10 interact with OMA1 to inhibit OMA1 activity, the mitochondrial integrated response stress (mtISR), and mitochondrial fusion processing of OPA1. The double knockout of CHCHD2 and CHCHD10 triggers mtISR, whereas the single knockdown of CHCHD2 promotes ISR [96]. In addition, missense mutations of OMA1 have been found in sporadic ALS patients, but more ALS patients need to be sequenced to determine whether OMA1 plays a role in ALS pathogenesis [97].

### 4.5. The Role of mtDAMPS in the ALS Inflammation

Neuroinflammation is a common feature of ALS. When tissues are damaged and invaded by infectious agents, the immune defense system responds rapidly, but may eventually lead to neuronal damage due to the persistent production of toxic inflammatory mediators such as cytokines, ROS, and reactive nitrogen species. Mitochondrial Damage Associated Molecular Patterns (mtDAMPS) are endogenous molecules that are often sequestered by the body’s cells as risk factors. Microglia are the main mediators of neuroinflammation, responsible for phagocytosing and removing dead cells, protein aggregates, other particles, and soluble antigens that threaten the central nervous system. mtDAMPS will be recognized by the immune receptors of microglia after activation. In addition, the activated mtDAMPS mediates the expression of inflammatory mediators and also trigger an intracellular cascade to regulate mitochondrial metabolism and function [98]. TNF is a pleiotropic cytokine involved in many chronic inflammations. Recent studies have provided compelling evidence that TNF and its downstream signaling pathways impair OXPHOS, and that the activation of TNFR1 can lead to the expression of many different cytokines, ultimately leading to apoptosis or programmed cell death. In addition, the expression of mitochondrial fission protein FIS1 was found to be increased in the mitochondria of 3T3-L1 adipocytes differentiated by TNF treatment, but the expression of the fusion protein OPA1 was decreased [99]. These results directly or indirectly indicate that pro-inflammatory cytokines such as TNF can affect mitochondrial dynamics in ALS. mtDNA is a key signaling molecule that triggers inflammatory response signals. A growing number of studies have found that immune-activated mtDNA and mtRNA can induce an aberrant production of pro-inflammatory cytokines and interferon effectors. The integrity of mitochondrial membranes is compromised following cellular stress or mitochondrial damage, when mtDNA is released from mitochondria into the cytoplasm, triggering aberrant pro-inflammatory and type I interferon (IFN) responses. However, it is worth noting that the use of VDAC1 oligomerization inhibitor VBIT-4 reduces mtDNA release and the inflammatory response, which may provide a potential therapeutic approach for ALS [100].

## 5. Ca^2+^ Dysregulation in ALS

Mitochondria are regulators of intracellular Ca^2+^ movement. When mitochondria take up Ca^2+^, it can promote mitochondrial metabolism and OXPHOS, thereby adjusting mitochondrial performance [101,102,103]. Mitochondria further influence electrophysiological activity through somatic dendritic formation as well as axonal and presynaptic Ca^2+^ oscillations [30]. Under basal conditions, the concentration of Ca^2+^ in mitochondria and cytoplasm is the same, approximately 100–200 nM. Mitochondria regulate Ca^2+^ concentration through voltage-dependent anion-selective channel proteins (VDACs), which promote Ca^2+^ from the matrix into the mitochondrial intermembrane space, and through the Ca^2+^ uniporter complex into the matrix [104].

Therefore, one of the features of impaired MNs in ALS patients is that Ca^2+^ homeostasis is disturbed [105], which has been simulated using in vitro or in vivo models with mutated genes such as SOD1 [106], TDP-43 [107], FUS, etc. In mitochondria, one of the main functions of the endoplasmic reticulum (ER) is to regulate the uptake of Ca^2+^, especially to promote Ca^2+^ exchange with mitochondria after the release of Ca^2+^ stored in ER, but TDP-43 disrupts the ER/mitochondria association, reducing mitochondrial Ca levels [108]. Interestingly, upon the activation of α-amino-5-methyl-3-hydroxyisoxazolone-4-propionic acid (AMPA) receptors, the recovery of physiological Ca^2+^ concentrations in MNs is delayed [109]. Because the high expression of AMPA receptors at postsynaptic terminals greatly reduces their intrinsic Ca^2+^ buffering capacity, MNs in ALS are thought to die due to Ca^2+^-induced excitotoxicity [110]. Consequently, MNs are particularly dependent on proper Ca^2+^ buffering by mitochondria [111].

Ca^2+^ exchange is regulated through the interaction of vesicle-associated membrane protein-associated proteins B and C (VAPB) with the mitochondrial protein, protein tyrosine phosphatase-interacting protein 51 (PTPIP51). Evidence suggests that VAPB disrupts Ca^2+^ homeostasis and disrupts mitochondrial anterograde axonal transport, which is critical for neuronal health and survival [112]. Mutations in VAPB suggest that disturbances in Ca^2+^ homeostasis are associated with familial ALS [113]. In addition, TDP-43 has been shown to disrupt the VAPB-PTPIP52 pathway; a similar mechanism is thought to exist in cases of idiopathic ALS, where pathogenic TDP-43 accumulates. At the same time, the decreased expression of VAPB in the spinal cord of ALS patients also supports this contention. A recent study showed that increased calcium permeability of AMPA and *N*-methyl-D-aspartic acid receptor (NMDA) is associated with insufficient mitochondrial Ca^2+^ uptake and reduced calcium buffering capacity due to an imbalance between MCU1 and MCU2 [114]. Thus, glutamate excitotoxicity in ALS is usually due to an imbalance in the MCU complex, resulting in defective mitochondrial Ca^2+^ buffering. However, another recent study showed specific alterations in the Ca kinetics of MNs in ALS patients, calling for different treatment strategies [115]. Table 1 summarizes proteins associated with impaired mitochondrial dynamics in ALS.

## 6. Biomarkers Associated with Mitochondrial Dysfunction in ALS

**Figure 3:**
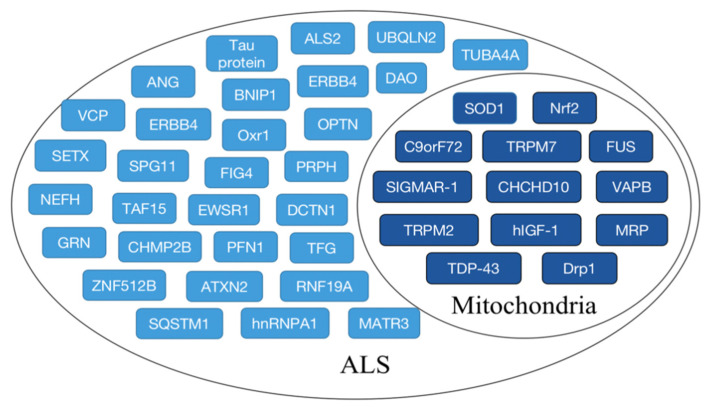
ALS-related biomarkers. The main biomarkers associated with ALS are shown, and biomarkers associated with mitochondrial dysfunction in ALS are indicated by dark blue boxes.

Mutations in genes can lead to mitochondrial fragmentation, repair defects, and morphological abnormalities in ALS patients, but the lack of precise gene targets hinders ALS treatment. Identifying signaling pathways and corresponding biomarkers is critical for discovering new treatments. Findings have identified a number of potential genetic biomarkers for clinical diagnosis, as shown in Figure 3.

The discovery of these markers provides a basis for in-depth research on the pathogenesis, targeted diagnosis, and treatment of ALS. Over the years, scientists have carried out mechanistic studies to clarify the impact of major markers on the organism after damage, as shown in Table 2.

## 7. Research Progress in ALS Treatment

Antioxidant therapy for mitochondria may be a major direction for future research involving targeted therapy drugs. Peroxisome-promoting life-activating receptor γ (PPARγ) is a ligand-activated transcription factor that can regulate mitochondrial function, maintain normal mitochondrial turnover, stabilize the redox balance, antioxidant response, immunity reaction, and fatty acid oxidation, etc. [125]. In SOD1-G93A mice, treatment with pioglitazone, an agonist of the PPARγ receptor, prolongs the survival of diseased mice, reduces gliosis [126], and protects MNs from p38-mediated neuronal death [127]. Tetramethylpyrazine nitric acid (TMP), a potent free radical-scavenging nitroso moiety, was found to reduce spinal motor neuron loss and glial cell responses after intraperitoneal injection in already diseased SOD1-G93A mice. This suggests that the antioxidant activity of mitochondria is activated by treatment with TBN, a potential targeted drug [128].

Furthermore, in a Drosophila ALS model based on the binding protein TDP-43, pioglitazone resolved TDP-43-dependent synergistic motor dysfunction in the MNs and glia but not in muscle [129]. Because pioglitazone may cause certain features of rhabdomyolysis, such as muscle pain, elevated phosphocreatine kinase, and weakness, which can worsen the symptoms of ALS. Hydroxocobalamin attenuated TDP-43 toxicity, reduced OS and mitochondrial dysfunction, and a combined treatment with a low-sugar diet significantly attenuated lifespan shortening and motor deficits in flies expressing TDP-43, suggesting that oral hydroxocobalamin may be a TDP-43-based therapeutic intervention for ALS [130]. Secondly, studies have shown that after mitoquinolmesylate (MitoQ) treatment of SOD1-G93A mice, the decline in mitochondrial function in the spinal cord and quadriceps muscle was slowed, and spinal cord nitrification markers and pathological symptoms were significantly reduced. Muscle junctions were restored, and lifespan was significantly extended in mice, suggesting that antioxidant targeting of mitochondria may play a pharmacologic role in the treatment of ALS, and clinical trials are underway [131].

The magnitude of mitochondrial membrane permeability is also critical for the regulation of Ca^2+^ buffering capacity. GNX4728, a regulator of mitochondrial membrane permeability, increases mitochondrial calcium retention by mitochondrial permeability transition pore (mPTP) [132]. Olesoxime, such as GNX4728, also works by regulating mPTP. Studies have demonstrated that olesoxime binds to two outer membrane proteins, voltage-dependent anion-selective channels, and translocator protein (TSPO), to alter the pore size of mitochondrial membranes, thereby reducing neuronal cell viability due to damage impact [133]. In animal experiments, it was found that olesoxime significantly reduces the death rate of MNs in ALS mice and improves the activation ability of microglia [134]. Since olesoxime belongs to the family of cholesterol oxime compounds, its toxicity was tolerated in the first two phases of clinical trials, but in the third phase clinical trial due to its too high toxicity, the experiment was declared to fail [135].

SigMar-1 is a highly conserved transmembrane protein that is selectively and highly expressed in MNs of the spinal cord and usually aggregates specifically on the endoplasmic reticulum membrane. As an agonist of the SigMar-1 receptor, keratin stabilizes mitochondria-associated membrane domains by regulating calcium flux, while reducing ROS generation by affecting PI3K-AKT signaling, thereby ensuring stability in neurodegenerative disease states such as ALS Supplies mitochondrial bioenergy [136].

We found that stem cells from human exfoliated deciduous teeth-conditioned medium (SHED-CM) significantly inhibited the intracellular aggregation and neurotoxicity induced by mutant SOD1 in ALS. Further, while it is protective against induced iPSC-derived MNs, the most important finding was that it is effective against both familial and sporadic ALS. These results suggest that SHED-CM is a potential therapeutic approach to slow the progression of ALS [137]. HEXA-018, a novel autophagy inducer, can increase the LC3-I/II ratio and increase the number of autophagic lysosomes. It was found that treatment with HEXA-018 significantly reduced damage to the ubiquitin–proteasome system and oxidative stress-induced neurotoxicity. This also suggests HEXA-018 as a new therapeutic candidate for related neurodegenerative diseases such as ALS [138]. Stem cell transplantation also provides an opportunity for neurotrophic factors to enter the nervous system and remodel areas affected by neurodegenerative diseases. However, issues such as the type and number of cells transplanted remain to be optimized [139].

Table 3 summarizes potential therapeutic targets for improving mitochondrial function in ALS and drugs effective for mitochondrial function therapy.

## 8. Conclusions and Outlook

ALS is the third most common adult-onset neurodegenerative disease [143], for which there is no cure or treatment. It destroys MNs and skeletal muscles, eventually paralyzing the patient and causing death [144]. While numerous experimental studies on ALS have been conducted worldwide, the exact mechanisms of disease onset and progression remain unclear. There are many reasons for the slow progress of research, including an unclear pathogenesis, a rapid disease progression, and a lack of suitable animal models [145]. The crucial role of mitochondria in meeting the physiological requirements of eukaryotes has attracted a great deal of attention to the chain reaction caused by mitochondrial dysfunction. Several mechanisms and genes have been firmly associated with mitochondrial dysfunction. Therefore, a better understanding of the pathogenic mechanism and the interlinkage between the various factors leading to mitochondrial dysfunction are needed to ensure more effective treatments [146].

Mitochondria are highly dynamic double-membrane subcellular organelles whose OXPHOS generates ATP and helps control metabolism [96]. The regulatory mechanism of mitochondria is extremely important in the process of maintaining cellular homeostasis. Factors such as genetic mutations or altered mitochondrial dynamics are triggers or facilitators of neurodegeneration. Therefore, the selective vulnerability of MNs in ALS may be related to mitochondrial numbers, mutated genes, and external influences.

As previously shown, the treatment of mitochondrial dysfunction has mostly focused on antioxidant, mitochondrial membrane pore regulation and signaling pathway intervention, and has made significant progress. However, clinical trials based on such research still face many challenges, including the identification of specific mutational loci, the regulation of signaling pathways, drug-induced neurotoxicity, and suboptimal therapeutic effects. To date, various targeted drugs have entered clinical trials, such as intravenous injection of the free radical scavenger edaravone 60 mg/day to ALS patients. After 6 months of treatment, the ALSFRS-R score of the patients decreased significantly. Edaravone has been approved for the treatment of ALS in the United States, Japan, Canada, Switzerland, and South Korea, but has not been approved in the European Union [147]. Masitinib is an inhibitor of tyrosine kinases. With concomitant riluzole treatment at a dose of 4.5 mg/day as an add-on, masitinib was also found to reduce ALSFRS-R score [148].

In general, after decades of research and clinical trials, the research on ALS and mitochondrial dysfunction has grown in depth and thoroughness, but there is an urgent need to find new specific biomarkers and achieve clinical translation of research results. Mechanism-specific drug development and combination therapies may become the main new avenues for treatment.

## Figures and Tables

**Table 1:** Proteins associated with impaired mitochondrial dynamics in ALS.

Protein	Change	References
Mitochondrial fission		
SOD1 TDP-43	Drp1 and Fis1 protein levels ↑ (mitochondrial fission ↑)	[40,45,46,47]
Mitochondrial fusion		
SOD1 TDP-43	Mfn1 and Opa1 protein levels ↓ (mitochondrial fusion ↓)	[40,46,47]
Mitochondrial degradation		
OPTN	Accumulation of damaged mitochondria	[49,50,51]
P62	Impaired LC3 recognition (Autophagy ↓)	[52,53]
Proteins related to disturbed mitochondrial Ca^2+^ handling		
TDP-43	Decreased contacts between mitochondria and ER (mitochondrial Ca^2+^ uptake ↓)	[109]
VAPB	Disturbed Ca^2+^ homeostasis	[112]

**Table 2:** Genetic markers associated with mitochondrial dysfunction in ALS.

Protein	Location/Coding Sequence	Result of Malfunction
SOD1 [116]	IMS	Mutated SOD1 induces ALS mitochondrial toxicity.
C9orf72 [117]	Non-coding region (GGGGCC)	Poly(GR) in C9ORF72-related ALS impairs mitochondrial function and increases oxidative stress and DNA damage in iPSC-derived MNs.
TDP-43 [118]	TARDBP (chromosome Ip36.2)	Mutant TDP-43 disrupts mitochondrial dynamics, and overexpression of TDP-43 results in abnormal mitochondrial aggregation and loss of normal function, resulting in progressive neuronal loss.
FUS [119]	Nucleus	FUS plays a role in a cascade of nuclear loss of function and increased cytoplasmic functional toxicity in ALS. Furthermore, FUS mutation and subsequent mislocalization to the cytoplasm sequester additional nuclear proteins critical for RNA metabolism, such as motor neuron proteins (SMN), blunting the nuclear activity of these proteins.
VAPB [120]	ER	VAPB depletion induces increased autophagic flux and decreased ATP production, thereby disrupting neuronal ion homeostasis and function.
SigMar-1 [121]	MANs (mitochondria-associated endoplasmic reticulum membranes, MAMs)	Regulates Ca^2+^ signaling between ER and mitochondria and maintains MAMs structural integrity.
Nrf2 [122]	Leucine zipper transcription factor	Dysfunctions in the Nrf2 result in a loss of redox homeostasis, leading to overload with reactive oxygen/nitrogen species.
TRPM7 [123]	Plasma	TRPM7 isoforms cause oxidative stress by inducing hypoxia-activated cation currents that increase ROS production.
hIGF-1 [124]		Deletion of hIGF-1 induces mitochondrial apoptosis, inhibits normal mitochondrial mitotic phagocytosis, and promotes motor neuron apoptosis.

**Table 3:** Treatments based on improving mitochondrial function in ALS.

Targets\Drugs	Result of Malfunction	Therapeutic Directions\Therapeutic Efficacy	References
Grxs	The verexpression of Grxs1 in IMS may accelerate mitochondrial fragmentation.	The overexpression of Grx2 interferes with mitochondrial fragmentation, preserves mitochondrial function, and protects neuronal cells from apoptosis.	[40]
OPTN and TBK1	The loss of OPTN or TBK1 function results in impaired mitochondrial phagocytosis and the accumulation of damaged mitochondria.	The binding of OPTN to the ALS-associated E478G ubiquitin prevented stable binding of the mutant to the mitochondrial surface. Furthermore, the recruitment of OPTN and LC3B to damaged mitochondria was significantly reduced using ALS-associated TBK1 mutants.	[50]
C9orf72	C9orf72 haploinsufficiency destabilizes mitochondrial complex I and drives motor neuron degeneration.	Maintaining the integrity of mitochondrial morphology protects C9orf72 from damage, which in turn reduces neuronal degeneration.	[53]
mtDNA	The accumulation of mtDNA mutations leads to increased oxidative damage, decreased energy production, and increased ROS.	Controlling the amount of ROS production and improving the correctness of replication and repair mechanisms may be a potential therapeutic mechanism.	[77]
Nrf2	The loss of Nrf2 accelerates motor neuron death and astrocyte activation, leading to early onset of the disease.	Mutations in the SOD1 gene lead to reduce Nrf2, and finding ways to reduce or inhibit SOD1 gene mutations may improve these problems.	[93]
TDP-43	The accumulation of TDP-43 can promote the increase in endoplasmic reticulum stress level, which in turn promotes the activation of apoptosis.	Calcium ions interrupt fine-tuned signaling between the ER and mitochondria and initiate apoptotic signaling cascades, thus serving as a convergence point for multiple upstream perturbations of cellular homeostasis and constituting a potentially important therapeutic target.	[95]
VBIT-4	mtDNA is a key signaling molecule that triggers inflammatory responses.	Reduced mtDNA release and inflammatory response with VDAC1 oligomerization inhibitor VBIT-4 may offer a potential treatment for ALS.	[100]
Hydroxocobalamin	TDP-43 toxicity impairs mitochondrial function.	Hydroxocobalamin attenuated TDP-43 toxicity, decreased OS and mitochondrial dysfunction, and combined treatment with a low-sugar diet significantly improved motor deficits, suggesting that oral hydroxocobalamin may be a TDP-43-based therapeutic intervention for ALS method.	[130]
MitoQ	In SOD1-G93A mice, mitochondrial function was significantly decreased in spinal cord and muscle, and spinal cord nitrification markers and pathological symptoms were significantly increased.	MitoQ treatment of SOD1-G93A mice slowed the rate of decline in mitochondrial function in the spinal cord and quadriceps, restored muscle connectivity and significantly increased lifespan in mice.	[131]
GNX4728	Changes in mitochondrial membrane permeability affect Ca^2+^ buffering capacity, which in turn affects mitochondrial metabolism and OXPHOS.	GNX4728 is a regulator of mitochondrial membrane permeability and increases mitochondrial calcium retention via mPTP.	[132]
SHED-CM	Mutations in the SOD1 gene are neurotoxic and induce intracellular aggregation.	Stem cells from SHED-CM can significantly inhibit the intracellular aggregation and neurotoxicity induced by mutant SOD1, have a protective effect on MN, and can be considered as a potential therapeutic approach to slow down the progression of ALS.	[137]
HEXA-018	OS induces neurotoxicity.	HEXA-018 increased the LC3-I/II ratio and increased the number of autophagolysosomes, while also significantly reducing damage to the ubiquitin-proteasome system and oxidative stress-induced neurotoxicity. This suggests that HEXA-018 could be a candidate for ALS treatment.	[138]
Respiratory chain complex	The inhibition of respiratory chain complex activity results in increased ROS production and decreased ATP production.	Cysteine peptide rT1 can promote ATP synthase and cell survival by targeting ETC.	[140]
Double-stranded DNA deaminase toxin A (DddA)	The mutation of mtDNA causes normal mitochondria to gradually die, resulting in abnormal mitochondrial function.	DddA potentially corrects highly pure and specific pathogenic mutations in mtDNA, a highly innovative therapeutic approach.	[141]
triphenylphosphine cation (TPP)	Oxidative stress increases ROS production, leading to cellular damage and decreased ETC activity.	TPP can effectively scavenge ROS and reduce oxidative stress, while also transporting functional proteins into mitochondria.	[142]

## Data Availability

Not applicable.

## Abbreviations

ALS	amyotrophic lateral sclerosis
OS	oxidative stress
MNs	motor neurons
SOD1	superoxide dismutase 1
TDP-43	transgenic binding protein 43
FUS	fused in sarcoma
MATR 3	matrin 3
CHCHD10	coiled-coil-helix-coiled-coil-helix domain containing 10
TBK1	tank-binding kinase 1
TUBA4A	tubulin, alpha 4A
C21orf2	chromosome 21 open reading frame 2
C9orf72	chromosome 9 open reading frame 72
CCNF	cyclin F
OXPHOS	oxidative phosphorylation
ROS	reactive oxygen species
NADH	nicotinamide adenine dinucleotide
FADH2	reduced flavin adenine dinucleotide
ETC	electron transport chain
IMM	inner mitochondrial membrane
MCU	mitochondrial calcium uniporter
OMM	outer mitochondrial membrane
IMS	inter membrane space
Grxs	glutaredoxins
DPR	dipeptide repeat
TIMMDC1	translocase of inner mitochondrial membrane domain containing 1
Fis1	mitochondrial fission protein 1
Mfn1	mitofusin 1
Drp1	dynamin-related protein 1
Opa1	optic atrophy 1
OPTN	optineurin
LIR	LC3-interacting region
SQSTM1	Sequestosome 1
H_2_S	hydrogen sulfide
CAT	catalase
PUFAs	polyunsaturated fatty acids
mtDNA	mitochondrial DNA
Nrf2	nuclear factor erythroid 2-related factor 2
ARE	antioxidant response element
VDACs	voltage-dependent anion-selective channel proteins
ER	endoplasmic reticulum
AMPA	α-amino-5-methyl-3-hydroxyisoxazolone-4-propionic acid
VAPB	vesicle-associated membrane protein-associated proteins B and C
PTPIP51	protein tyrosine phosphatase-interacting protein 51
NMDA	*N*-methyl-D-aspartic acid receptor
MANs	mitochondria-associated endoplasmic reticulum membranes
TRPM7	transient receptor potential cation channel subfamily M member 7
hIGF-1	human insulin-like growth factor 1
PPARγ	peroxisome-promoting life-activating receptor γ
TMP	Tetramethylpyrazine nitric acid
MitoQ	Mitoquinolmesylate
mPTP	mitochondrial permeability transition pore
TSPO	translocator protein
SHED-CM	stem cells from human exfoliated deciduous teeth-conditioned medium
